# Notes and News

**Published:** 1888-10-06

**Authors:** 


### Notes and News.

Collected and Communicated.

The first meeting of The Hospitals Association will be held at St. Thomas's Hospital on Wednesday, 10th inst., at eight p.m. A paper will be read on the "History, Position, and Prospects of the National Pension Fund for Nurses," by Mr. E. T. Clifford, Honorary Manager of the Fund. The chair will be occupied by Dr. T. Syer Bristowe, F.R S., Senior Physician to the Hospital and President of the Association.

Mrs. Cutts, of Belfort House, Harrogate, has given £1,000 to the Beckett Hospital, Barnsley, in memory of her parents, Mr. and Mrs. Leadman.

The late Mr. Daniel Thwaites, formerly M.P. for Blackburn, left £10,000, through his daughter, Mrs. Yerburgh, wife of the member for Chester, to the Blackburn and East Lancashire Infirmary.

The patients in the Currie Wards of the London Hospital have expressed their thanks, through the columns of the *Standard*, to those persons who were kind enough to send them some chessmen and boards, in reply to an appeal made there.

The enlightened people of Wakefield have been raising money for the Clayton Hospital by means of open-air concerts of sacred music held on Sunday afternoons. The sixth of these has been given with great success, there being almost 300 performers, and 15,000 of an audience. The takings amounted to £73 0*s.* 4*d.*—a miserably small sum to be received from so many people.

A Gordon Memorial Window was unveiled in the Manchester Cathedral after morning service, on Saturday, by the Very Rev. the Dean, in the presence of Canon Kelly, Canon Woodhouse, the Dean of Ely, Mr. Maclure, M.P., and a large number of clergymen and laymen. The window is the gift of Mr. C. J. Schofield, J.P., and is the work of Messrs. Wilson and Whitehouse, of Vernon Place, Bloomsbury Square, London.

The Hospital Saturday Fund is £800 in excess of the amount received up to the corresponding date last year. Workshop and other collections will continue to be received until the end of November. It is expected the final total will largely exceed that of any former year.

A meeting of representatives of the various friendly societies in Richmond and neighbourhood, convened by Mr. G. C. Rowland, secretary to the Richmond Hospital, was recently held in the Lecture Hall, Hill Street, Richmond, to consider the advisability of holding a Hospital Saturday demonstration in aid of the funds of that institution. It was agreed to hold this on the 20th inst., when it will be sought to raise money by means of collecting-boxes, and by the sale of flowers.

In aid of the funds of the Alexandra Hospital for Sick Children, a two days' sale of work was inaugurated on the 26th ult. in the Board Room. The financial condition of the hospital is now a subject of some anxiety to the committee, and the ladies who have organised the bazaar are to be commended for the trouble they have taken towards helping to clear the debt now existing. The sale of work is not a very extensive one, as may be gathered from the fact that it is confined to the Board Room, but it is hoped it will do much to improve the hospital funds.

Considering that the importance of providing for the safe treatment of persons suffering from diphtheria must have long been known, it is surprising that the other day, when the medical officer for the parish of St. Pancras "telephoned to Norfolk House," in a case of great urgency, the reply was: "No accommodation for diphtheria anywhere in London." It seems incredible that no provision should have been made for the reception of cases of a disease at once so infectious and so common.

A severe blow has been dealt by the trustees of the Manchester Royal Infirmary to the system of hospital physicians and surgeons accepting appointments at more than one institution. At a special meeting of the trustees on Monday last the following resolution was passed almost without a dissentient voice: "That no assistant physician or surgeon appointed after the 17th September, 1888, shall, after becoming a physician or surgeon, hold any other hospital appointment."

Sims Reeves has nearly completed fifty years of public life. That his voice should still be a delight to hear speaks volumes for the value of exercise in the preservation of the vigour of a physiological organ. A "Life" of the famous singer has just been published by Messrs. Simpkin, Marshall, and Co. The great man is his own biographer. He tells us that his first operatic triumph was won in Milan, and that he might have secured the highest success in Italy, that land of singers, had he not found it both more congenial and more profitable to follow his fortunes in his own country. His countrymen owe him no grudge for the choice he made.

All London was startled and horrified on Sunday by the announcement that two more murders had been committed at the East End on Saturday night. There is reason to believe that one or both of them were the work of the same hand that had been guilty of the death of Annie Chapman. We discuss elsewhere the reported offer of a high price for certain physiological organs made by an American. Whether there be any connexion at all between the offer and the murders there is nothing as yet to show. That such a connexion is quite possible, those who are familiar with the story of Dr. Knox's dealings with Burke and Hare will be only too ready to admit.

The following vacancies are announced this week, the amount of salary in each case being given after the name of the institution :—

**Resident Medical Officers.**

Brompton Hospital for Consumption. House Physician.
City of London Chest Hospital, Victoria Park. Resident Clinical Assistant.
Dorchester County Asylum. Assistant Medical Officer.—£120 per annum, with board, etc.
Essex Lunatic Asylum. Junior Assistant Medical Officer.—£100 per annum, with board, etc.
Liverpool (Royal Southern) Hospital. Junior House Surgeon. —Sixty guineas, with board, etc.
Salop Infirmary, Shrewsbury. Assistant House Surgeon.—Board, etc.
St. Olave's Union, Bermondsey. Resident Medical Officer.—£300 per annum.
Sheffield Public Hospital and Dispensary. Junior Assistant House Surgeon.—£50, with board, etc.
Weston super Mare Hospital and Dispensary. House Surgeon. —£60 per annum, with board, etc.

**Non-resident Medical Officers.**

Bermondsey Ves'ry. Analyst.—Fee 10s. each analysis.
Bristol Dispensary. Medical Officer.
Central London Throat and Ear Hospital, Gray's Inn Road. Three Clinical Assistants.
Dental Hospital of London, Leicester Square. Lecturer on Dental Surgery and Pathology.

---

The Mayor has issued his customary appeal to the clergymen and ministers of religion of Birmingham in connexion with the Hospital Sunday collections which are to be made on October 28th on behalf of the following institutions : The General Dispensary, the Children's Hospital, the Eye Hospital, the Women's Hospital, the Royal Institution for the Deaf and Dumb, the Institution for the Blind, the Homœopathic Hospital, the Orthopædic and Spinal Hospital, the Birmingham Sanatorium, the Lying-in Charity, the Skin and Lock Hospital, the Ear and Throat Infirmary, the Dental Hospital, and the District Nursing Society. This is the thirtieth year since the commencement of united efforts on the part of the congregations of Birmingham to assist the local charities. The appeal urges that if possible every other appeal for help private to congregations may stand over in order that one united and cordial effort may be made by all who worship God in behalf of a cause which knows no denomination—the relief of suffering humanity.

---

There is perhaps no work undertaken by kindly-disposed persons more necessary or more useful than that performed by the "After-Care Association." It deals with convalescents who are recovering from attacks of insanity. Who is so much to be pitied as the insane ? Who demands, and ought to command, such tender and thoughtful care during convalescence ? The mind that is delicately balanced may be moved from its equilibrium by the most trifling circumstance. If this be so in health, what must be the added risk when an acute attack has just been experienced ? The association has sent us a copy of its annual report, which we gladly notice. During the past year forty-eight cases have been before the committee, of which forty-one have been accepted. Most of these cases have been sent to country cottages for a season, in Kent, Surrey, Sussex, Essex, and Somerset. The system of boarding-out these convalescents in separate cottages is found far more successful and satisfactory than that of bringing them all together in one large home. The latter method is practically the continuance of asylum life under another name. It is gratifying to report that in nearly every case where convalescents have, after a period of cottage life, been placed in service. they have given complete satisfaction, and no relapse has followed. Her Royal Highness the Princess Christian is the patroness of the society, the Earl of Meath is the president, and Mr. Thornhill Roxby the secretary. The bankers are the Union Bank of London, Regent Street Branch.

F. L. May and Co.'s newspaper advertisement offices have been removed from 159, Piccadilly, to their new premises on the ground floor, 162, Piccadilly (corner of St. James's Street, W.).

---

At the Royal Lunatic Asylum, Montrose, where the new buildings so long contemplated are now in course of erection, gangs of insane patients may be seen at work, digging at foundations, and otherwise applying themselves to skilled and unskilled labour. This question of work for the insane has received much attention in Scotland, and the best results have followed. In no country are the mentally afflicted treated with more care, intelligence and success than across the border. The new buildings at Montrose, when completed, will cost nearly £13,000. The work is being to a large extent carried out by the employés of the asylum, assisted by the patients.

---

Mr. Francis J. G. Mason, M.R.C.S., has been elected to the office of resident surgeon at the Cheltenham Branch Dispensary, in succession to Mr. W. R. Buckell. Mr. Mason has held a position for nearly four years past in connexion with the General Hospital—two years as junior, and the remainder of the time as senior house surgeon. There were nearly forty applications for the vacant post, and of the three candidates selected by the House Committee for an interview with the Hospital Board, Mr. Mason was ultimately elected by a large majority. The election of Mr. Mason is a well deserved acknowledment of the satisfactory manner in which his duties at the hospital have been discharged.

---

The Queen's Jubilee did not prove pecuniarily advantageous to every hospital in the United Kingdom last year, though it helped the funds of some. Among the fortunate few was the Clayton Hospital at Wakefield. An old-standing debt was cleared off, and extensive repairs were undertaken and paid for, the total amount raised for these purposes being the handsome sum of £1,501. The Saturday Fund fell off by £73 as compared with last year, and this loss was principally due to the diminished amount collected at the open-air concerts. Fifteen thousand people attended one of the concerts, and only a little over ten thousand coins were received, showing that nearly five thousand had passed the gates, enjoyed the music, and paid—nothing. Are the colliers and factory hands at Wakefield "hard up," or do "dog-running" and "cock-fighting" demand too much of their "loose cash," as they did twenty years ago ?

---

The sixty-first annual meeting of the Salford and Pendleton Royal Hospital was held at the Town Hall, Salford, on the 26th ult. Mr. Shelmerdine, hon. treasurer, read the annual report, from which it appeared that the number of persons under the care of the hospital during the past year was 14,081, divided into 3,656 home patients, 5,192 out-patients, 796 in-patients, and 4,437 accident patients. The financial position of the charity is one that causes anxiety to the committee, and requires immediate attention. The expenditure exceeded the income during the year just concluded by £1,586 1*s*. 9*d*. The committee therefore urge the importance of a largely increased subscription list, otherwise it may be necessary to close some of the wards. During the past year legacies of over £8,000 had been received, and had been placed in the investment account. The board expressed regret that Mr. Shelmerdine had felt compelled to retire from the post of treasurer and chairman of the board, which positions he had for a long time honourably and efficiently filled, and they had pleasure in announcing that Mr. George A. Southam had consented to undertake the duties of treasurer. Concluding, the board suggested that some memorial of the services of Mr. Shelmerdine should be provided.

